# Transformation and Toxic Effects of Pollutants in Agricultural Environment

**DOI:** 10.3390/toxics14030252

**Published:** 2026-03-12

**Authors:** Changbo Zhang, Liang Peng, Weijie Xue

**Affiliations:** 1 Agro-Environmental Protection Institute, Ministration of Agriculture and Rural Affairs, Tianjin 300191, China; zhangchangbo@caas.cn; 2 College of Environment and Ecology, Hunan Agricultural University, Changsha 410128, China

## 1. Introduction

The agricultural environment is of critical for human survival on Earth, especially as the water–soil–crop agricultural environment system is closely related to human health [1]. Pollutants such as heavy metals [2,3], microplastics, and pesticides [4,5,6] enter the agricultural environment through agricultural production, posing threats to agricultural safety and seriously affecting human health. Therefore, it is critical to explore the transformation and migration of pollutants in the agricultural environment and their toxicity changes and conduct research on related pollution remediation technologies to protect the quality of the agricultural environment.

## 2. An Overview of the Published Articles

Soil heavy-metal pollution poses a significant threat to soil quality and human health. Radina Nikolova et al. (contribution 1) evaluated the diversity and structural changes of microbial communities and assessed the ecological risks posed by different soils contaminated with heavy metals while predicting the key metabolic pathways related to soil microbial resistance. They emphasized the necessity of including soil microbial indicators in agricultural management strategies to ensure food safety. The impact of microplastics on the bioavailability of heavy metals is a current research hotspot. Zhenbo Wang et al. (contribution 2) conducted soil culture experiments to investigate the effects of polyethylene microplastic concentrations on soil properties, bacterial communities, surface chemical properties, and the distribution of forms of cadmium and lead in the soil, revealing that the concentration of polyethylene particles changed the physical and chemical properties of the soil and the structure of the bacterial community, ultimately affecting the transformation of forms of cadmium and lead.

Seeking to reduce the toxicity of heavy metals, Fu Lin et al. (contribution 3) conducted field experiments on double-cropping rice, explaining that spraying ionic liquids on the surfaces of the leaves of rice plants can inhibit the absorption, transport, accumulation, and toxicity of cadmium and arsenic in rice grains by promoting the synthesis of amino acids and regulating the absorption and transportation of essential elements. The results of field experiments conducted by Chen Kexin et al. (contribution 4) showed that spraying citric acid on the surfaces of rice plant leaves significantly reduced the soluble cadmium content in the plant’s organs and promoted the transformation of cadmium from soluble into non-soluble forms, thereby inhibiting the transport of cadmium from plant organs to rice grains. Ge Lei et al. (contribution 5) evaluated the key sites for controlling cadmium transfer in rice through leaf surface control technology and revealed the good blocking function of the endophytic microbial community biofilm with respect to the transport of heavy metals in rice. Zhang Shuo et al. (contribution 6) explored the potential of malic acid in reducing cadmium toxicity and investigated its genotype-dependent influence on cadmium absorption and essential element balance in rice. These findings provide a mechanistic basis for developing leaf-surface-conditioning agents based on micro-addition and formulating management strategies for heavy-metal pollution targeting different genotypes. These results provide technical support and a theoretical basis for reducing the excessive cadmium content in rice.

Soil pesticide residues constitute another widely concerning pollution problem. Jiale Zhang et al. (contribution 7) developed a method for microbially degrading new neonicotinoid insecticides in soil, studied the ability of Aspergillus versicolor in soil to degrade acetamiprid, and determined the optimal degradation conditions for and conducted toxicological analysis of acetamiprid and its metabolites. These findings will aid researchers in conducting safety assessments of the toxicological properties of neonicotinoid insecticides and their biodegradable metabolites, along with related studies on their degradation capabilities. Luan Gabriel Xavier de Souza et al. (contribution 8) investigated the adsorption of atrazine on pristine and aged polyethylene microplastics and evaluated its impact on plant toxicity. The results showed that the coexistence of microplastics and atrazine led to greater toxicity, indicating that the synergistic effect of pollutants can amplify the negative impact on plant development. Yuan Zhang et al. (contribution 9) reported that machine learning techniques are becoming increasingly valuable in simulating the transport of pollutants in plant systems and applied three symbolic regression models, based on original and enhanced data, to obtain accurate predictive equations. The results showed that machine learning models and symbolic regression methods can provide crucial insights into pollutants’ absorption and accumulation in plant roots.

## 3. Conclusions

This Special Issue focuses on the transfer process and influence mechanisms of agricultural environment pollutants in the water–soil–crop system and explores the toxicity changes in heavy metals, microplastics, pesticides, and perfluoroalkyl and polyfluoroalkyl substances in the soil during their transfer in agricultural systems. It reports on leaf surface control technology and the way it is used to reduce excessive heavy metal concentrations in rice, along with the screening effect of heavy metals on soil microbe species. It introduces a machine learning model designed to reveal the synergistic effects of microplastics to enhance their toxicity and explains the mechanism by which microplastics affect the bioavailability of cadmium and lead in the soil. In summary, the articles published in this Special Issue cover the behavior and processes of pollutants in the agricultural environment; the diffusion mechanisms of pollutants; the factors affecting the distribution, migration, and transformation processes in the soil–crop system; and health risk assessments in the agricultural environment.

## Data Availability

No new data were created or analyzed in this study. Data sharing is not applicable to this article.
